# Bayesian Multi-View Clustering given complex inter-view structure

**DOI:** 10.12688/f1000research.126215.1

**Published:** 2022-12-09

**Authors:** Benjamin D. Shapiro, Alexis Battle

**Affiliations:** 1 Department of Computer Science, Johns Hopkins University, Baltimore, MD, 21218, USA; 2 Department of Biomedical Engineering, Johns Hopkins University, Baltimore, MD, 21218, USA

**Keywords:** clustering, multi-view, Bayesian models, gene expression, methylation, multi-omics, public health

## Abstract

Multi-view datasets are becoming increasingly prevalent. These datasets consist of different modalities that provide complementary characterizations of the same underlying system. They can include heterogeneous types of information with complex relationships, varying degrees of missingness, and assorted sample sizes, as is often the case in multi-omic biological studies. Clustering multi-view data allows us to leverage different modalities to infer underlying systematic structure, but most existing approaches are limited to contexts in which entities are the same across views or have clear one-to-one relationships across data types with a common sample size. Many methods also make strong assumptions about the similarities of clusterings across views. We propose a Bayesian multi-view clustering approach (BMVC) which can handle the realities of multi-view datasets that often have complex relationships and diverse structure. BMVC incorporates known and complex many-to-many relationships between entities via a probabilistic graphical model that enables the joint inference of clusterings specific to each view, but where each view informs the others. Additionally, BMVC estimates the strength of the relationships between each pair of views, thus moderating the degree to which it imposes dependence constraints. We benchmarked BMVC on simulated data to show that it accurately estimates varying degrees of inter-view dependence when inter-view relationships are not limited to one-to-one correspondence. Next, we demonstrated its ability to capture visually interpretable inter-view structure in a public health survey of individuals and households in Puerto Rico following Hurricane Maria. Finally, we showed that BMVC clusters integrate the complex relationships between multi-omic profiles of breast cancer patient data, improving the biological homogeneity of clusters and elucidating hypotheses for functional biological mechanisms. We found that BMVC leverages complex inter-view structure to produce higher quality clusters than those generated by standard approaches. We also showed that BMVC is a valuable tool for real-world discovery and hypothesis generation.

## Introduction

In biomedicine, public health, and a number of other fields, an unprecedented availability of increasingly diverse large-scale data related through complex mechanisms, both known and unknown ( *e.g.* multi-omic patient information in combination with clinical and demographic data), has sparked the need for unsupervised machine learning methods that leverage both quantity and diversity of information. For many years, the field of machine learning has primarily emphasized questions of data abundance ( *e.g.* high dimensionality). However, approaches tailored to integrative analysis and multi-view learning have recently come into focus. By leveraging the structure shared between multiple views of the same system, we expect to improve the quality of data clustering in any given view. We are thus able to construct a more informed representation of the latent structure and any properties unique to a particular modality. Discovery of this kind can improve our understanding of both the high- and low-level functional mechanisms that describe the system. As the heterogeneity and complexity of data sources steadily increases, it is critical to scientific advancement that we conceive of more flexible and interpretable machine learning approaches to understanding diverse, multi-modal collections of related data.

Several useful frameworks for multi-view clustering exist to-date, but these share the assumption that the cross-domain relationships between entities are one-to-one, *i.e.* that each view measures the same set of units. One of the most utilized models in this class, iCluster ^1^ and its extensions ^2^^–^^4^ use a joint latent variable model to integrate all data types and produce a single integrated clustering. These consensus-focused approaches employ a variety of strategies to learn an integrated representation rather than individual clusterings of the views. In contrast, the Multiple Dataset Integration (MDI) approach ^5^ aims to learn individual clusterings at a view-per-view granularity while incorporating known dependencies. Bayesian Consensus Clustering ^6^ and Clusternomics ^7^ also produce individual clusterings and jointly infer explicit global structure. These approaches provide more insight into the underlying mechanisms and unique properties governing the structure in each view than might be available when using consensus-focused approaches but are limited by their one-to-one assumption.

Critically, the one-to-one assumption quickly breaks down in many applications where sets of entities differ across views. In these cases there may be a many-to-one or many-to-many correspondence between views, and certain entities may not have corresponding related entities measured across all domains. For example, we may want to cluster epigenetic data across the genome (view 1) alongside related gene expression (view 2) and protein level (view 3) measurements. Methylation profiles in particular typically consist of many more entities (probes) than gene expression profiles (genes) with multiple probes mapping to, or near, a single gene; in some cases, a probe may not correspond to any of the measured genes. A gene may likewise correspond to more than one protein product due to alternative splicing effects ^8^ or to no protein if the gene is not protein-coding. Across domains, one common solution to this problem is to condense each dataset in order to force a strict one-to-one correspondence between views by combining or discarding measurements via some heuristic. The first shortcoming of this approach is that it eliminates information across modalities thereby reducing power. Furthermore, it can lead to downstream models which fail to account for important inter-view relationships. In addition, the extent to which relationships between views should bias estimates of intra-view structure is not always obvious. As such, another critical task is to learn the degree to which clusterings should inform one another. These challenges motivate methods of multi-view clustering that can intelligently account for more complex relationships between views.

Most recent efforts to address these challenges rely on extensions of non-negative matrix factorization ^9^ or its multi-view variant multiNMF. ^10^ Co-regularized Graph Clustering (CGC) ^11^ poses the problem as a multi-view graph clustering and, as we do, assumes that the inter-view relationships are known *a priori.* Given a primary view of interest (the view “in focus”), CGC estimates the relative contributions of all other views to the overall clustering. Complex Mapping Multi-View Clustering (CMMVC) ^12^ takes a similar approach and additionally proposes a method for estimating unknown inter-view relationships as a pre-processing step. These approaches lack some of the flexibility and interpretability of probabilistic approaches like the model we propose here, and also require some additional assumptions such as choosing a primary view. Similar efforts have been to made to perform multi-view clustering in contexts where data is incomplete. Incomplete multi-view clustering algorithms (IMC) ^13^^–^^16^ often rely on matrix factorization, graph learning, kernel and/or deep approaches to construct a representation of the data from which a consensus clustering can be learned from the incomplete data. These solutions relate to part of the problem we pose here where relationships may be missing but not where relationships may extend beyond a one-to-one mapping. There may also be cases where an entity in one view may not be related to an entity in another view, but not due to missing data; in these cases, the assumption of incompleteness made by IMC is not always appropriate. Finally, it is again important to note that little to none of the existing work in this area has taken a fully Bayesian approach.

Here we propose Bayesian Multi-View Clustering (BMVC), an approach that uses a novel graphical model to jointly learn the strength of every pairwise relationship between views, along with a separate clustering within each view that takes into account user-supplied inter-view relationships. A key contribution of BMVC is that it is a Bayesian approach based on mixture models, similar to that used in MDI. ^5^ This flexible probabilistic framework allows users to model a variety of data types; quantify uncertainty; and incorporate a variety of assumptions by specifying priors on the mixture component distributions, the sparsity of component weights, and the weight of the inter-view dependency. The cluster base distribution used in BMVC may be chosen to fit key assumptions regarding the latent distribution of the data ( *e.g.* allowing Gaussian process component priors to model clusters of time series data). Most importantly, to the best of our knowledge, BMVC is the first Bayesian clustering approach to rigorously leverage complex inter-view relationships between entities – such as one-to-many, many-to-many or one-to-zero relationships – to improve clustering quality.

## Methods

### Model

**Figure 1.**
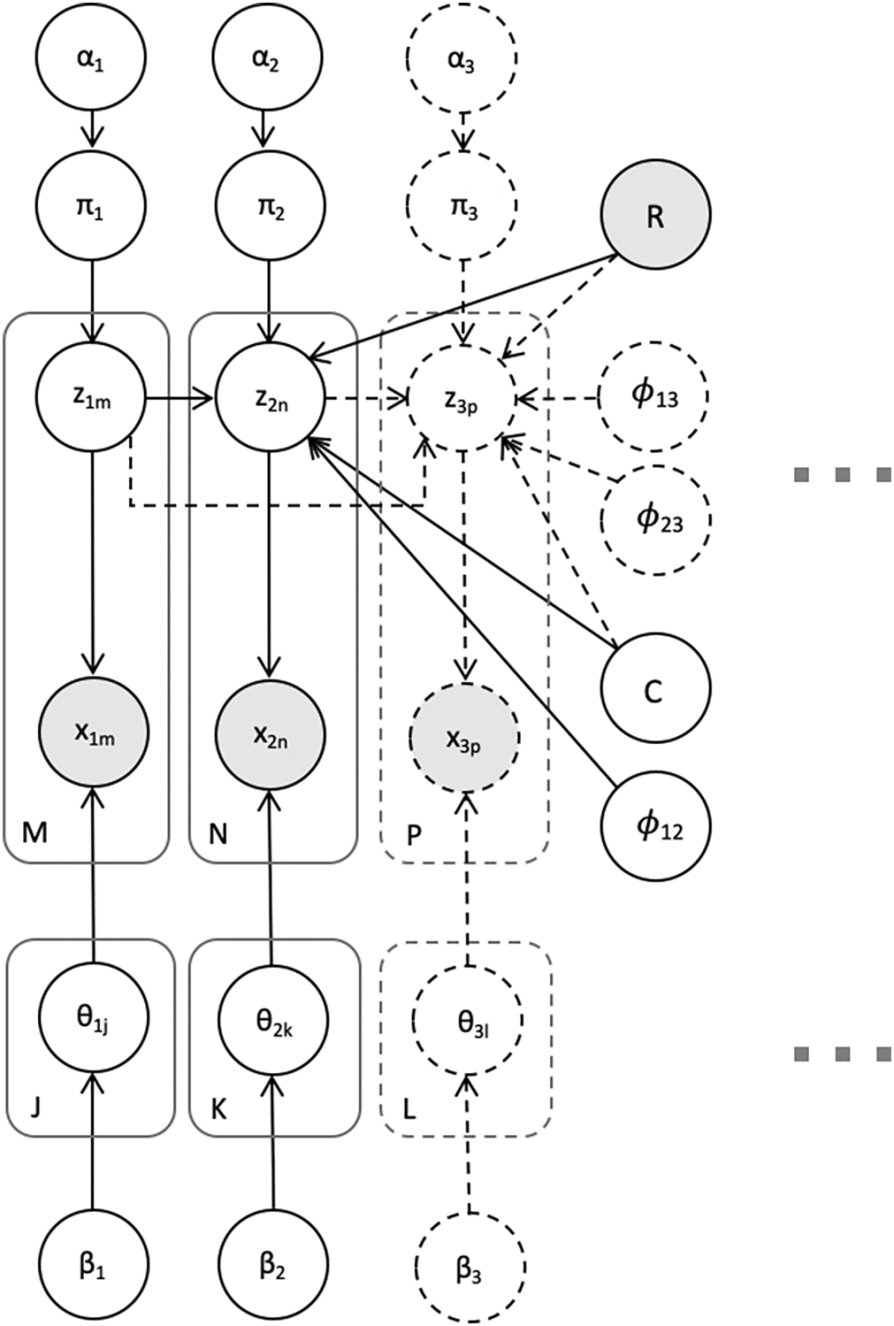
A plate-notated visualization of Bayesian Multi-View Clustering (BMVC) for an application with two views (shown in solid lines) as well as an extension to the three-view model (shown in dashed lines). Further views may be incorporated in a similar fashion. Gray nodes denote variables known *a priori.*

Let *v* ∈ 1… *V* index the corresponding view in a V-view clustering application, and let *k* ∈ 1… *K* index a cluster. For ease of notation, assume the same total number of clusters *K* across all views, although the full model permits specification of *K _v_* on a per-view basis. Note that even with fixed *K*, the true numbers of clusters learned in each view are not required to be the same or exactly *K*; they may be arbitrarily smaller if elements are assigned to only a subset of clusters and others are left empty in the most likely clustering found via inference. The BMVC model likelihood can be viewed as a modification of the joint likelihood of *V* independent mixture models with an additional term encouraging related entities to cluster similarly across views. The probabilistic graphical model is shown in plate notation in Figure 1.

Let *x* be the data, *G _v_* be the probability density function of the component distribution parameterized by *θ*, *z* be the component assignments and *π* be the *K*-dimensional vector of mixture weights. Further, we specify priors on the *θ* using a base distribution *G*_0_ parameterized by *β*, and Dirichlet priors on the mixture weights ( *π _v_*) parameterized by *α.* Let *v* and *i* respectively index the view and entities of the data such that *x _vi_* refers to entity *i* in view *v* and *z _vi_* refers to the corresponding cluster assignment. Assume the following prior distributions:

$$\begin{array}{c} \pi_{v}∼\mathrm{Dir}\left(\frac{\alpha_{v}}{k},\ldots \frac{\alpha_{v}}{k}\right)\\ \theta_{\mathrm{vk}}∼G_{0}\left(\beta_{v}\right)\\ z_{\mathrm{vi}}∼\text{Multi}\left(\pi_{v}\right)\\ x_{\mathrm{vi}}∼G_{v}\left(\theta_{{\mathrm{vz}}_{\mathrm{vi}}}\right) \end{array}$$

For simplicity, we denote the number of entities for view *v* as *N _v_.* Building up from a standard mixture model with no ties between views, the conditional joint probability of all the component assignments *Z* of *V* independent Gaussian mixture models is merely a product of their priors dictated by *π*:

$$p\left(Z|\pi \right)=\prod_{v=1}^{V}\prod_{i=1}^{N_{v}}\pi_{z_{\mathrm{vi}}}$$

In addition to the standard mixture model priors described above, we specify three additional variables *R*, *C* and *ϕ*:

• Let *R _vu_* be a known, user-specified indicator matrix that encodes the graph of related entities between two views indexed *v* and *u.* Note that *R* may also have continuous positive values that describe known relationship weights. Specifically, assume that *R _vu_* [ *i*, *j*] is non-zero if a relationship exists between entity *x _vi_* and *x _uj_.* For example, if the views respectively measure gene expression and methylation, then R should be non-zero where gene *i* is regulated by methylation probe *j.*
• Let *C _vu_* be a latent indicator matrix that encodes the graph of related matrices between two views indexed *v* and *u*, where every row and column has exactly one indicator. This reflects the assumption that every cluster in a view has a single corresponding cluster in another. Specifically, assume that *C _vu_* [ *k*, *l*] is non-zero if cluster *k* in view *v* relates to cluster *i* in view *u.*
• Let $\phi_{\mathrm{vu}}\in {\mathbb{R}}^{+}$ be a latent variable representing the overall strength of the relationship between views *v* and *u.*

### BMVC cluster assignments

We place a conditional joint prior on the traditional mixture model cluster assignments as follows:

$$\begin{array}{l} p\left(Z|\pi ,R,\phi \right)\propto \prod_{v=1}^{V}\prod_{i=1}^{N_{v}}\pi_{z_{\mathrm{vi}}}\cdot \\ \prod_{v=1}^{V-1}\prod_{i=1}^{N_{v}}\prod_{u=v+1}^{V}\prod_{j=1}^{N_{u}}\left(1+C_{\mathrm{vu}}\left[z_{\mathrm{vi}},z_{\mathrm{uj}}\right]R_{\mathrm{vu}}\left[i,j\right]\phi_{\mathrm{vu}}\right) \end{array}$$

Comparing the assignment strategy that results from this prior with that of the traditional mixture model, we see that BMVC increases the probability of a clustering assignment for a given entity if any related entity across views is assigned to a cluster with the same label. Note that the expression requires a normalization constant to define a distribution (as in a Markov random field). The normalization constant $\bar{Z}$ is computed by summing this quantity over all possible cluster assignments of all the entities across the views. Assume each view *v* ∈ 1… *V* is associated with a number of clusters *K _v_*, and a number of entities *N _v_*:

$$\begin{array}{l} \bar{Z}=\sum_{z_{11}=1}^{K_{1}}...\sum_{z_{1N_{1}}=1}^{K_{1}}...\sum_{z_{V1}=1}^{K_{V}}...\sum_{z_{{\mathrm{VN}}_{V}}=1}^{K_{V}}\;\\ \prod_{v=1}^{V}\prod_{i=1}^{N_{v}}\pi_{z_{\mathrm{vi}}}\cdot \\ \prod_{v=1}^{V-1}\prod_{i=1}^{N_{v}}\prod_{u=v+1}^{V}\prod_{j=1}^{N_{u}}\left(1+C_{\mathrm{vu}}\left[z_{\mathrm{vi}},z_{\mathrm{uj}}\right]R_{\mathrm{vu}}\left[i,j\right]\phi_{\mathrm{vu}}\right) \end{array}$$

### Model estimation

We perform approximate inference to jointly learn the optimal values for the cluster weights *π*, cluster distribution hyper-parameters *θ*, cluster assignments *z*, and the latent *ϕ.* To learn the model we conduct a modified hard expectation maximization procedure. In the E-step we compute the expected cluster assignments given the other model parameters. Note that *R* is comprised of connected components of related entities across views. All assignments are dependent within a connected component of *R*; thus, an exact estimate of the expected assignment of any entity with relationships requires considering all possible combinations of assignments to all entities in the corresponding component of the relationship graph. Given complex relationships across multiple views with many related entities, this computation can quickly become intractable. To address this complexity, we perform the E-step in an iterative, alternating fashion by greedily estimating the cluster assignments within each view conditioned on the assignments made in the others, effectively performing coordinate ascent.

The M-step is comprised of three sub-steps. We compute the analytical maximum likelihood estimates of the cluster distribution parameters *θ*, which are conditionally independent of the other parameters given the assignments chosen in the E-step. Next, we attempt to maximize the likelihood with respect to the cluster relationship matrices *C* by iteratively evaluating re-assignment of relationships between possible pairs of clusters across views. The inclusion of *C* in our model thus allows us to switch cluster dependencies during our inference procedure as we converge to clusterings where new cluster relationships form. Finally, we are able to use black box mean field variational inference (BBVI) ^17^ or a direct optimization via ADAM ^18^ to update the cluster weights and parameters *ϕ.* To reduce the variance of the BBVI gradient estimate and increase the efficiency of the method, we choose Gaussian variational distributions and utilize the reparameterization trick. ^19^

In the M step, it is necessary to estimate the normalization constant of the conditional prior on the cluster assignments. Unfortunately, as the number of clusters, views, and/or the size of the related groups of entities go up; this computation eventually becomes intractable. In these cases, we utilize the pseudolikelihood ^20^ to approximate the partition function tractably. The pseudolikelihood performs comparably to the full likelihood in all of our simulations.

The coordinate ascent algorithm for performing the E-step in conjunction with the expectation-maximization algorithm and the BBVI algorithm each guarantee local optima. As such, we employed random restarts to explore local optima and ultimately improve the quality of the results.

### Simulations

To test the efficacy of BMVC, we simulated a fully dependent two-view dataset where samples are drawn in each view from the same two independent clusters (A and B) defined by two-dimensional Gaussians. In this framework, related samples are generated by the same Gaussian distribution in each view. However, half the samples from A in the second view are shifted independently along the axis between the two cluster means, thus simulating data which are generated by cluster A but confounded by other effects in the second view. Such an effect might occur in a related modality with predictable underlying mechanistic differences. Let *x _A_* and *x _B_* be samples generated from *A* and *B* and let *s* be the shift percentage. We sample respectively:

$$\begin{array}{l} x_{\mathrm{Ai}}∼N\left(\mu_{A},\sigma_{A}\right)\\ x_{\mathrm{Bi}}∼N\left(\mu_{B},\sigma_{B}\right)\\ s_{i}∼N\left(\mu_{S},\sigma_{S}\right) \end{array}$$

To shift a sample *x _Ai_* closer to *μ _B_* we apply the following transformation:

$$\text{Shift}\left(x_{\mathrm{Ai}}\right)=x_{\mathrm{Ai}}+s_{i}\cdot \left(\mu_{B}-\mu_{A}\right)$$

**Figure 2.**
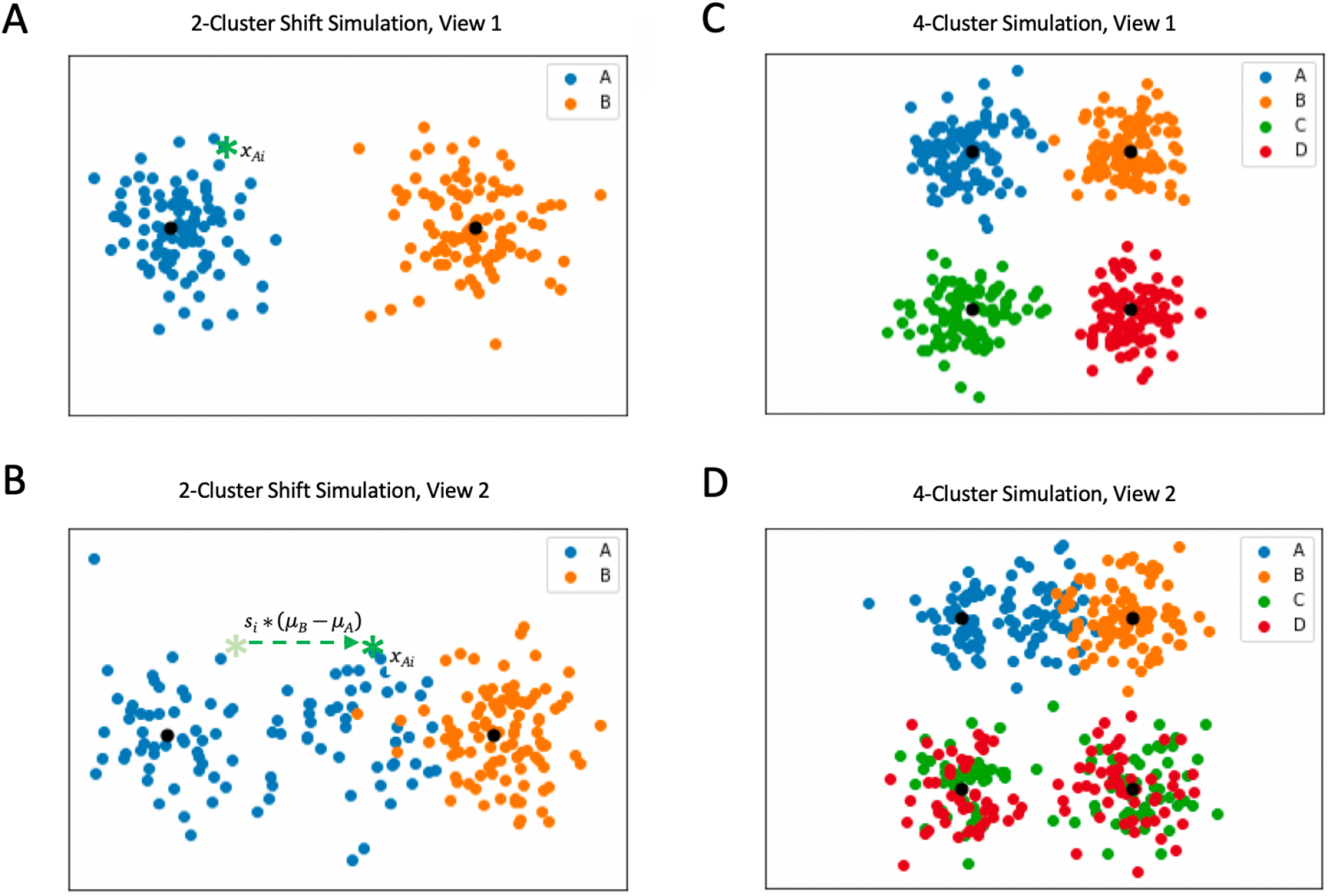
Visualizations of simulations with different types of confounding. A: The first view of an example simulation with 100 samples from each cluster. B: Depicts the shift in view 2 dictated by variable *s _i_* for sample *x _i_* (shown as a green star in the first view). Means of the Gaussians used to generate the data are depicted in black. C: Shows the first view of an example 4-cluster simulation. D: Demonstrates two types of confounding added in the second view (a shift and a binomial randomization effect) for use in comparing BMVC to the naive consensus clustering.

Thus, sample membership inference is confounded in the second view due to the proximity of some of the *A* samples to *B* (see Figure 2, left). We varied the shift degree *s* and tested our ability to recover the correct clustering in both views given three structural cases: a semi-missing one-to-one simulation in which half the samples of view 2 have one-to-one relationships to samples in view 1; a complete one-to-one case without missingness; and finally a two-to-one simulation in which view 2 has twice as many samples, which map in pairs to individual view 1 samples. We compared Gaussian BMVC to a baseline model with *ϕ* set to zero; this produced two independent mixture models optimized with respect to hard cluster assignments (H-GMM).

In order to test our ability to estimate *ϕ* given varying levels of dependence between views, we adapted a previous approach to introduce randomness into the dependence of the cluster assignments. ^7^ This was accomplished by re-assigning samples which would normally belong to cluster *A* some of the time per a binomial distribution parameterized by *p* chosen between 0 and 0.5. Within this framework, a baseline cluster *A* sample is generated by cluster *A* with probability *p* and is otherwise generated by cluster *B* with probability 1 − *p*; likewise, a cluster *B* sample is generated by cluster *B* with probability 1 − *p.* Thus, a *p* closer to 0.5 will reduce the dependence between views. We constrained the degree of relatedness of the datasets by introducing this modification into the data generated in the second view and observed our estimation of *ϕ* as we reduced the relatedness of the datasets.

### Naive consensus clustering

In order to explore the performance of BMVC in comparison to a baseline, multi-view approach, we generated a more complex four-cluster simulation with similar elements of confounding. In addition to original simulation clusters *A* and *B*, we generated data from two additional clusters *C* and *D.* In view 2, we confounded these randomly such that half of the cluster *C* samples in view 2 were related to cluster *C* samples in view 1, and half were related to cluster *D* samples in view 1. Similarly, half of the cluster D samples in view 2 were related to cluster *C* samples in view 1 rather than cluster *D* samples. Figure 2 (right) demonstrates the ground truth clusters of view 1 and their confounded relatives in view 2. Additionally, we introduced the same shift-based confounding as used previously between clusters *A* and *B* in view 2. We compared BMVC clusterings of these data to the naive consensus clustering produced by concatenating these two datasets along one dimension and applying a single Gaussian mixture model.

## Results

### Simulation results

**Figure 3.**
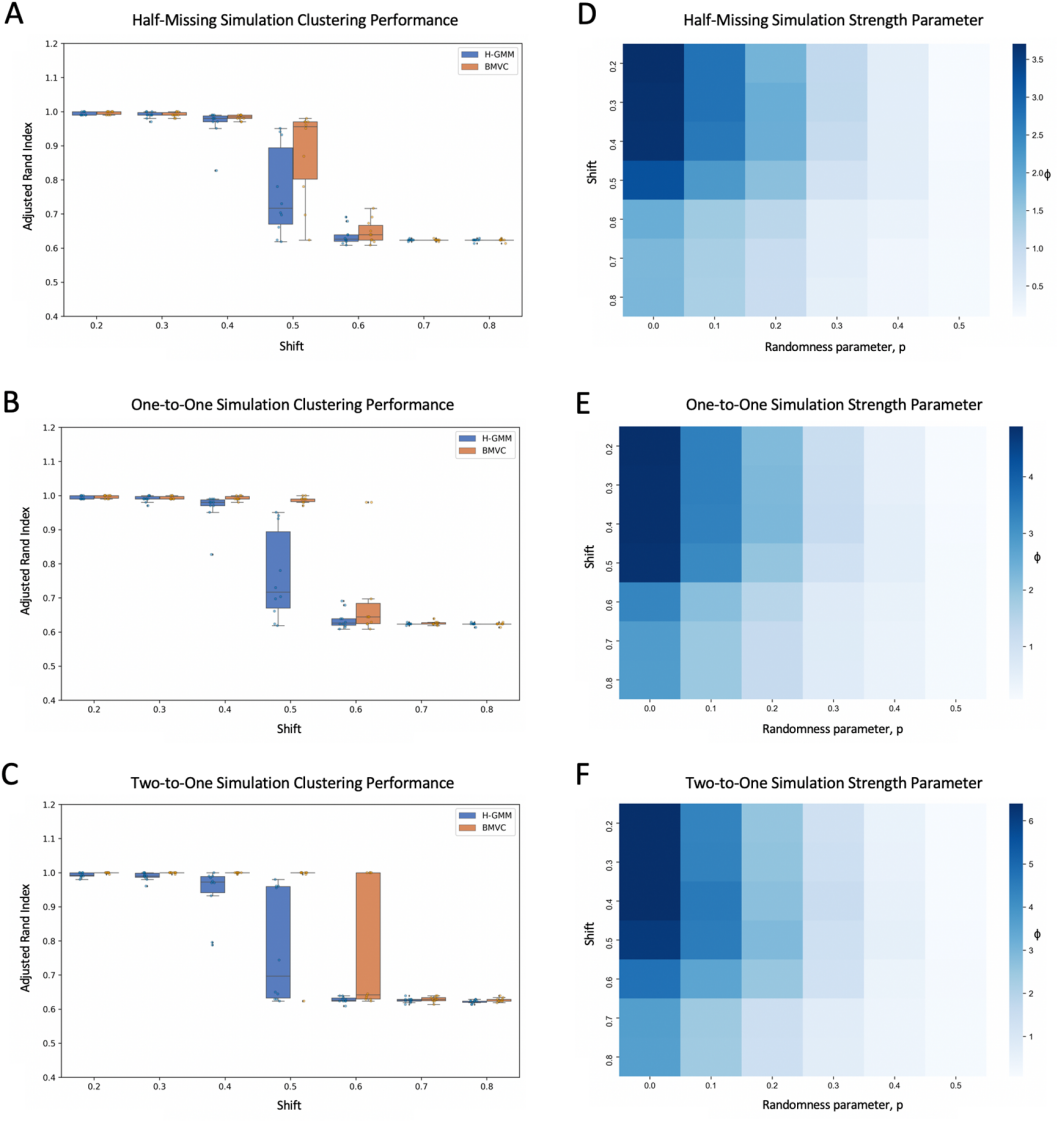
Results of applying Bayesian Multi-View Clustering to 2-cluster simulations. On the left, we show the adjusted Rand index of simulations with one-to-one half-missing relationships (A), one-to-one complete relationships (B), and two-to-one relationships (C) over varying degrees of shift. Solid lines represent the median score and demonstrate the improvement of BMVC over H-GMM. To the right, we plot the corresponding BMVC estimates of the dependence strength parameter *ϕ* over varying degrees of shift and view independence *p.*

To measure the quality of our simulations, we computed the adjusted Rand index (ARI) of the clustering generated by the model in comparison to the ground truth of the simulation and computed the average of this statistic over both views. The adjusted Rand index measures the similarity between two clusterings while accounting for chance groupings of the elements. We ran each simulation ten times and Figure 3 shows the results of each trial. As expected, BMVC and the baseline model (H-GMM) performed comparably at either end of the spectrum of shift degrees. When the shift was negligible, the true clustering was easily recoverable in view 2 without leveraging view 1. When the shift was too great, the shifted samples became so confounded with cluster *B* that we were no longer able to reasonably separate them. When shifts were substantial but did not significantly overlap cluster *B*, BMVC was able to leverage the known relationships between the two views to determine the correct cluster memberships, whereas H-GMM was not able to make such a distinction. Notably, the margin in improvement increased considerably as we added relationships in the complete one-to-one simulation ( Figure 3, middle) and then doubled the structure again in the two-to-one case ( Figure 3, bottom).

Figure 3 also shows the strength parameter learned by BMVC over combinations of the binomial parameter *p* and shift distance. There is a clear effect of the artificial randomness injected by *p.* As expected, BMVC estimated a higher dependence strength for simulations in which p was lower, and, thus, the inter-view dependence was more complete. This effect was stronger when there were more inter-view relationships.

**Figure 4.**
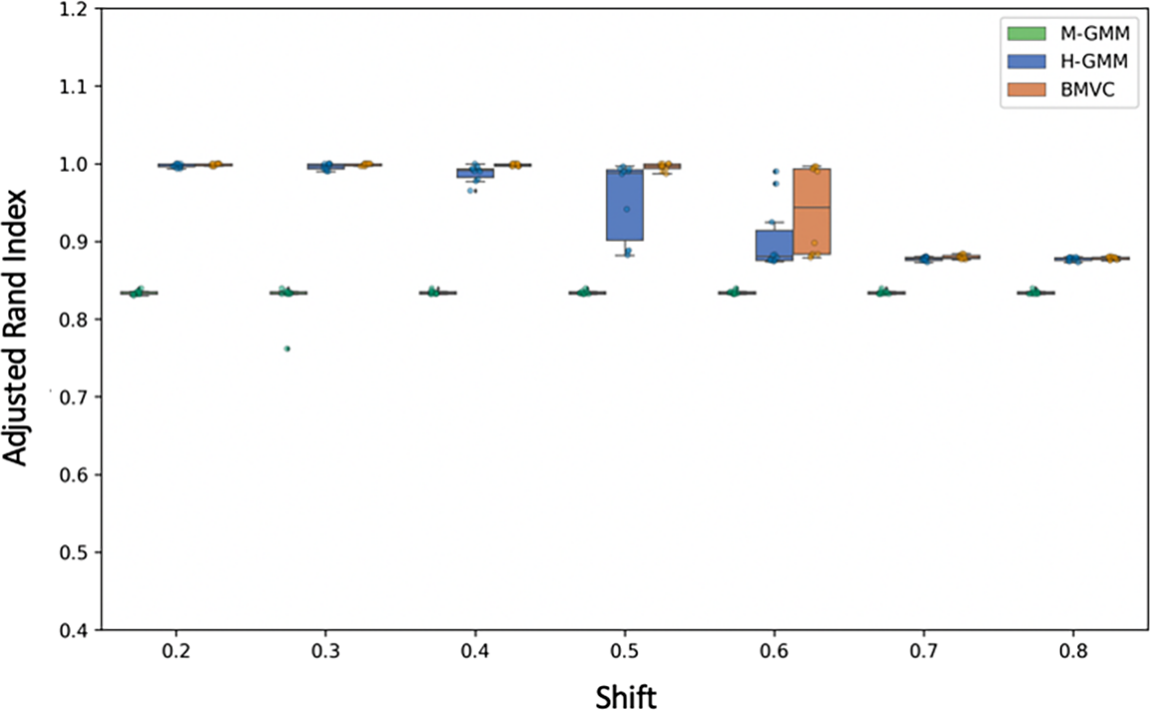
ARI of Bayesian Multi-View Clustering compared to a naive consensus clustering in the 4-cluster simulation.

In our 4-cluster simulations with two types of confounding, we found that both baselines (individual clusterings [H-GMM] and the naive consensus clustering [M-GMM]) produce insufficient interpretations of the data. The individual clusterings were unable to effectively deal with shift-based confounding while the naive consensus clustering approach was unable to capture the different structures present in the two views. In contrast, BMVC leveraged the known relationships to separate the shift-based confounding while also allowing for separate interpretations of the remaining data in the two views. Figure 4 demonstrates the improvement of BMVC over the two other approaches in terms of ARI.

### Application to public health outcomes in Puerto Rico following Hurricane Maria

**Figure 5.**
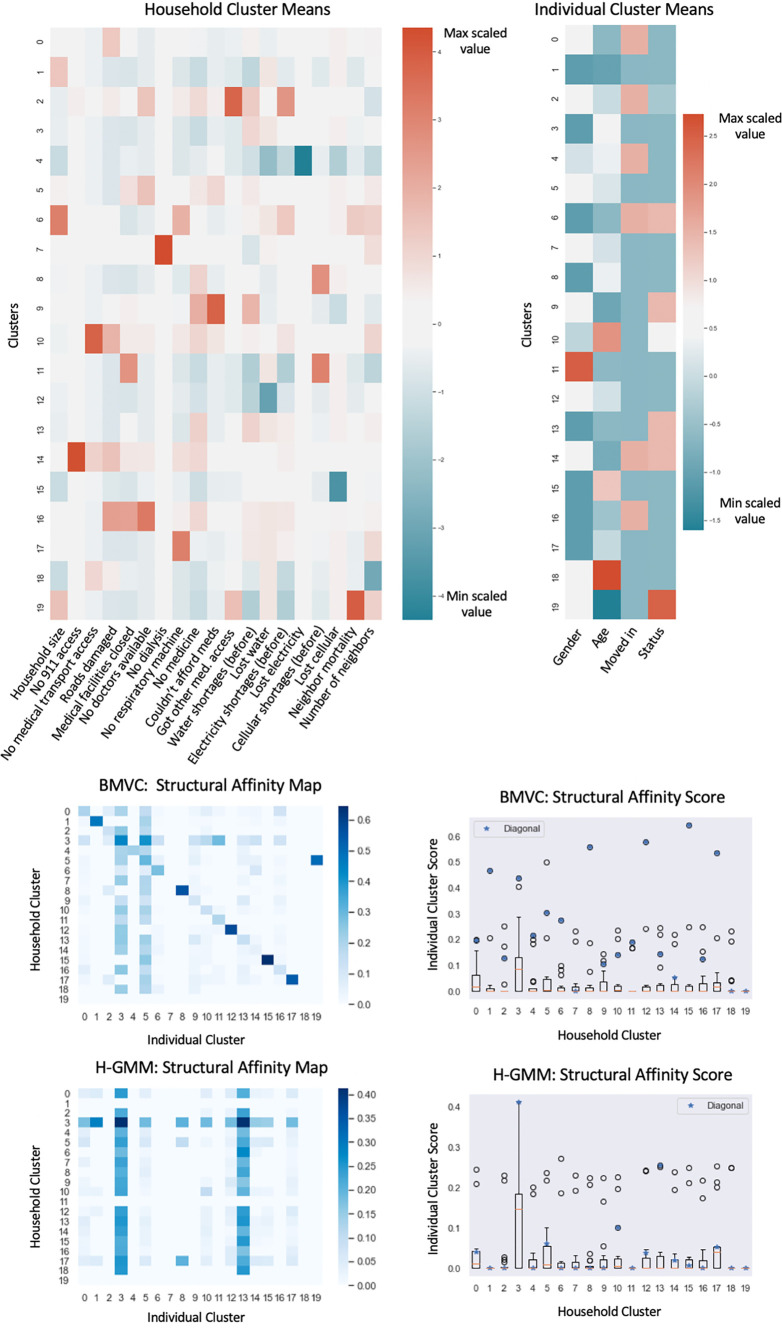
Results of applying Bayesian Multi-View Clustering to the households and individuals in the Puerto Rico dataset. Scaled means of the BMVC clusters of households and individuals in the Puerto Rico dataset are shown at the top. Features are scaled across clusters in each view to ease the interpretation of the feature importances of each cluster relative to the others. Structural affinity maps (bottom) are shown alongside score box plots that depict affinity scores along each map row with blue stars denoting the score of the matched cluster.

To examine BMVC’s ability to leverage inter-view relationships and its utility for real-world problems, we first turned to an easily interpretable application in public health. We applied BMVC to survey data collected from 3,299 households and 9,522 individuals, ^21^ which details access to various resources along with individual demographics and statuses of residents of Puerto Rico following Hurricane Maria. For the purpose of clustering, features in both views (individual-level and household-level) were filtered to feature sets common to all samples. The data includes general information such as household size and barrio, as well as information regarding the impact of the hurricane on households such as loss of access to medical facilities/equipment, loss of electricity, or loss of water. Individual data includes age, gender, whether the individual moved into their home that year, and their current status. Table 1 provides a heuristic guide to categorical feature values visualized in Figure 5. BMVC is ideally suited to fully leverage the multi-view structure present in the data because household size, and thus the degree of many-to-one relationships, varies from one individual to as many as 13.

**Table 1.** Categorical variable guide.

Variable	Range
Gender	Low: feminine; high: masculine; highest: other
Moved in	Low: no; high: yes
Status	Low: no change; med: died in 2017; high: left and did not return; highest: missing

### Individual and household cluster analyses

To increase the interpretability of the experimental results, we ran a sample BMVC clustering on the data using a prior on the *ϕ* parameter which favors highly related clusters across the views ( *i.e.* prefers large *ϕ*; all exact parameters of the run can be found in the provided Github repository). We found that the high degree of many-to-one relationships in the data made optimization of the full model likelihood intractable; as such, we utilized the pseudo-likelihood during inference. To assist in our analysis of the resulting clustering, we computed the proportion – for every pair ( *A* and *B*) of inter-view clusters – of the relatives of cluster *A* entities that are in cluster *B* and of the relatives of cluster *B* entities that are in cluster *A.* The average of these statistics (hereafter referred to as’structural affinity’) represents the degree to which the learned clusters adhere to the structural assumptions coded by *R.*

Figure 5 shows the cluster means (top) produced by BMVC as well as the structural affinity scores (bottom) of the BMVC clustering of the data and the hard-GMM clustering of the data for comparison. Structural affinity tends to be maximal along the diagonals much more often for the BMVC clustering than for the H-GMM, which demonstrates that the model successfully biases related entities to cluster more often under the paired labels encoded by *C* in contrast to two standard independent clusterings. Notably, clusters in the independent H-GMM household clustering do not tend to have uniquely high affinity scores with any particular individual clusters apart from the largest clusters (individual clusters 3 and 13), which are inherently more likely to contain individuals related to a given household. As such, the independent clustering of the data did not demonstrate many observable trends across views. In contrast, inspection of the cluster pairs with the highest structural affinity along the diagonal in the BMVC affinity map demonstrates that the model accurately captures and integrates some fundamental relationships between household conditions and individual demographic trends along with some additional, more complex causal relationships between household conditions and individual status ( Table 2).

**Table 2.** Household and individual clusters demonstrating structural affinity.

Clust	Household AVG.	Indiv AVG.
1;3;8;15	Larger households; no outlier events	M, younger/older, uneventful status
5;12	Less water outage; no outlier events	F, younger/older, uneventful status
17	Lost resp. mach. access	M, older, uneventful status
6	Widespread issues, known neighbor deaths	M, younger, left
2	Sought help at temporary aid center	F, slightly older, some deaths

Cluster labels for which we estimated the lowest structural affinity across views in this clustering demonstrate the unique properties of the individual views in cases where the latent structure is not related across the views; thus, these represent a form of negative control for inter-view structure. For example, individual cluster 19 does not demonstrate structural affinity across the views and corresponds to missing individuals. Household cluster 18 does not demonstrate structural affinity across the views and corresponds to households where members reported significantly fewer neighbor deaths than the average. Household cluster 7 describes households which lost access to dialysis for an average of 2.25 days and does not demonstrate structural affinity across the views in this clustering, which suggests that such cases may not have significantly contributed to mortality in this study. Previous findings suggest that end-stage renal failure typically results in death after an average of seven days in hospice patients without dialysis. ^22^ This could explain why the clustering does not reflect a significant relationship between this medical issue and a mortality event or relocation.

Intuitively, we noted a correlation between rarer negative outcomes and lower structural affinity, which might reflect smaller sample sizes and the less deterministic relationships between worsening household conditions and individual outcomes. For example, household cluster 2 describes households that were more likely to require assistance at a temporary aid center and demonstrates only moderate structural affinity to a cluster containing slightly older individuals and some deaths. Household cluster 9 describes households where access to medical care was limited by affordability and has even less structural affinity to an individual trend given by this clustering.

Finally, we turn to an examination of the clusters with the highest structural affinity across views. Because half the features in the individual view are purely demographic, households with relatively few adverse events cluster alongside various demographic groups of individuals with uneventful status ( *i.e.* that did not move or die) such as in clusters 1, 3, 5, 8, 12; further, households with widespread issues with electricity, water, cell availability, and that reported known deaths of neighbors were more likely to cluster alongside younger individuals who left despite having recently moved in (cluster 6). However, we are also able to make more specific observations about the relationships between very particular household factors and the individuals in those households; for example, households that reported a loss of access to respiratory machinery due to power failure (CPAP, BiPAP or nebulizers) clustered strongly alongside men averaging middle aged (cluster 17), coinciding with previous findings that sleep apnea and COPD are more likely to be diagnosed and to occur in older men. ^23^^,^^24^ This is an example of the way in which BMVC can be used for mechanistic hypothesis generation.

### Application to methylation accompanying prognostic gene expression in breast cancer patients

We now turn to a two-view genomic data application in which we clustered gene expression and methylation. This gave us the opportunity to more rigorously test BMVC’s ability to both improve clustering interpretability and increase clustering quality as measured by biological homogeneity.

Using patient data from The Cancer Genome Atlas (TCGA), ^25^ we examined a set of 1,819 genes identified as intrinsically related to breast cancer subtype in four different studies of breast cancer patients and further studied by TCGA. We selected 1,363 methylation probes which fell within the gene bodies of the intrinsic genes according to available Infinium HumanMethylation27 BeadChip annotations ^26^ and which also fell into the top 50% of probes with the highest variance over the 466 patients for which data was available across all’omics platforms. Before filtering to the selected genes and probes, we mean-centered and scaled each subject in each view over all loci. A total of 366 genes in our target set mapped to more than one probe in the high variance set, and 1,019 of them were unmapped. Of the probes, 23 were related to more than one gene. As such, this biological application includes both many-to-one relationships and missing relationships that are crucial to fully understanding the available structure. Before clustering, we performed principal component analysis on the full dataset and removed the first principal component in each view of the data as a conservative measure to account for potential technical artefacts. We then projected the data in each view onto the top 10 principal components – accounting for the highest 41% and 30% of the remaining variances in the expression and methylation views respectively – to avoid any cluster quality issues related to dimensionality. Consequently, we clustered the genes and methylation probes each in 10-dimensional space.

To test the capability of BMVC to produce biologically homogeneous clusters in the presence of confounding signal, we designed a preliminary experiment wherein we produced two dependent pseudo-simulated views from the gene expression data by independently adding standard Gaussian noise in each view. We then duplicated 25% of the entities at random in one of the views in order to simulate many-to-one relationships. Finally, we removed 25% of the edges from the resulting true relationship graph to simulate cases where some entities have no known relationships across views. We ran twenty initializations of BMVC with a Gaussian base distribution on this data alongside H-GMM and compared the Adjusted Rand Index (ARI) of the H-GMM and BMVC clusterings relative to a single-view H-GMM clustering of the original expression data without noise (henceforth referred to as the ‘ground truth’ GMM or GT-GMM) in order to determine whether BMVC is able to leverage the artificial inter-view structure to recover signal closer to the latent ground truth despite the inclusion of the random signal. We evaluated the biological homogeneity of the clusterings in terms of the similarity of the genes in each cluster using a similarity metric derived from the pairwise Gene Ontology Term Overlap (GOTO) ^27^ statistic, which is similar to that used by. ^5^ The GOTO score of a pair of genes is computed by retrieving Gene Ontology (GO) terms and their ancestors for each gene and estimating the pairwise intersection; thus, higher scores indicate a stronger relationship between gene products. We average these scores over pairs within a cluster. Then, we take a weighted average of the cluster scores over the clusters (where cluster sizes are the weights). We computed GOTO scores in each of three GO categories (Biological Process, Molecular Function, Cellular Component) as well as with respect to all GO terms regardless of category.

As in our preliminary experiment, we ran BMVC on the true TCGA data using a Gaussian base distribution. We evaluated the quality of the clusterings in contrast to H-GMM cluster labels for twenty random initializations. For methylation probes with multiple related genes, we estimated a GOTO score by averaging over all possible gene pairs. This process is further detailed in an included GitHub repository (see Extended data).

Finally, we ran Gene Set Enrichment Analysis (GSEA) ^28^ on the resulting gene expression clustering. GSEA is known to be biased when applied to methylation data, ^29^ but we were able to interpret much of the learned structure due to the natural cluster alignment produced by BMVC’s inter-view dependence.

### Analysis of biological results

**Figure 6.**
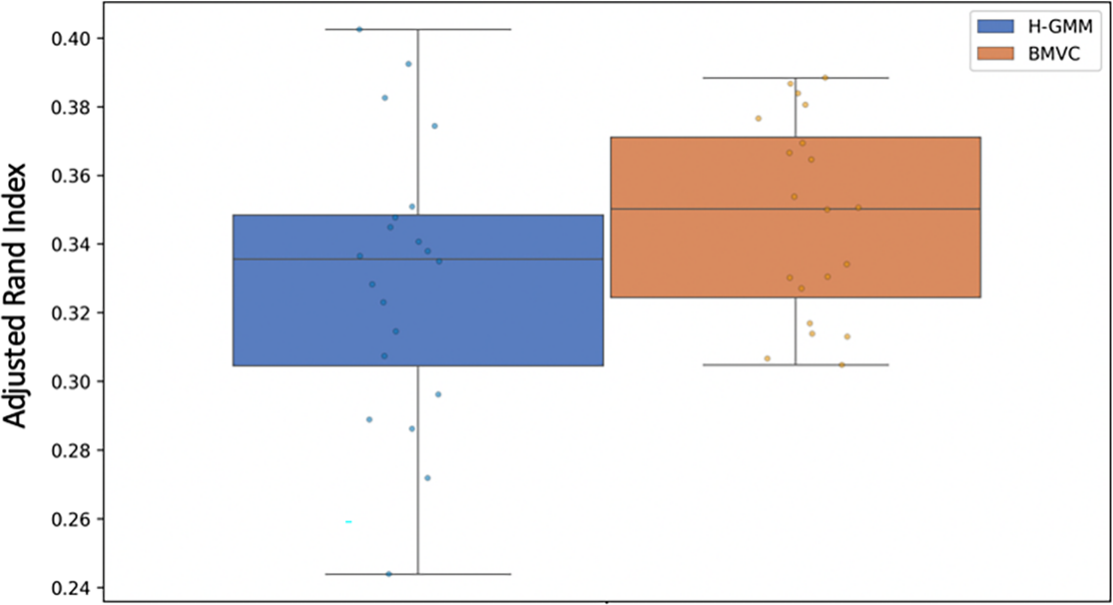
Adjusted Rand index of 20 Bayesian Multi-View Clustering (BMVC) clusterings alongside hard-assignment GMM (H-GMM) clusterings of pseudo-simulated data in the first view. The H-GMM clustering of the true expression data was considered to be ground truth for the purpose of ARI computation. BMVC captured true underlying biological structure more accurately than H-GMM on average. ARI was only computed in the first view due to the lack of ground truth in view 2 caused by duplication of the data.

**Figure 7.**
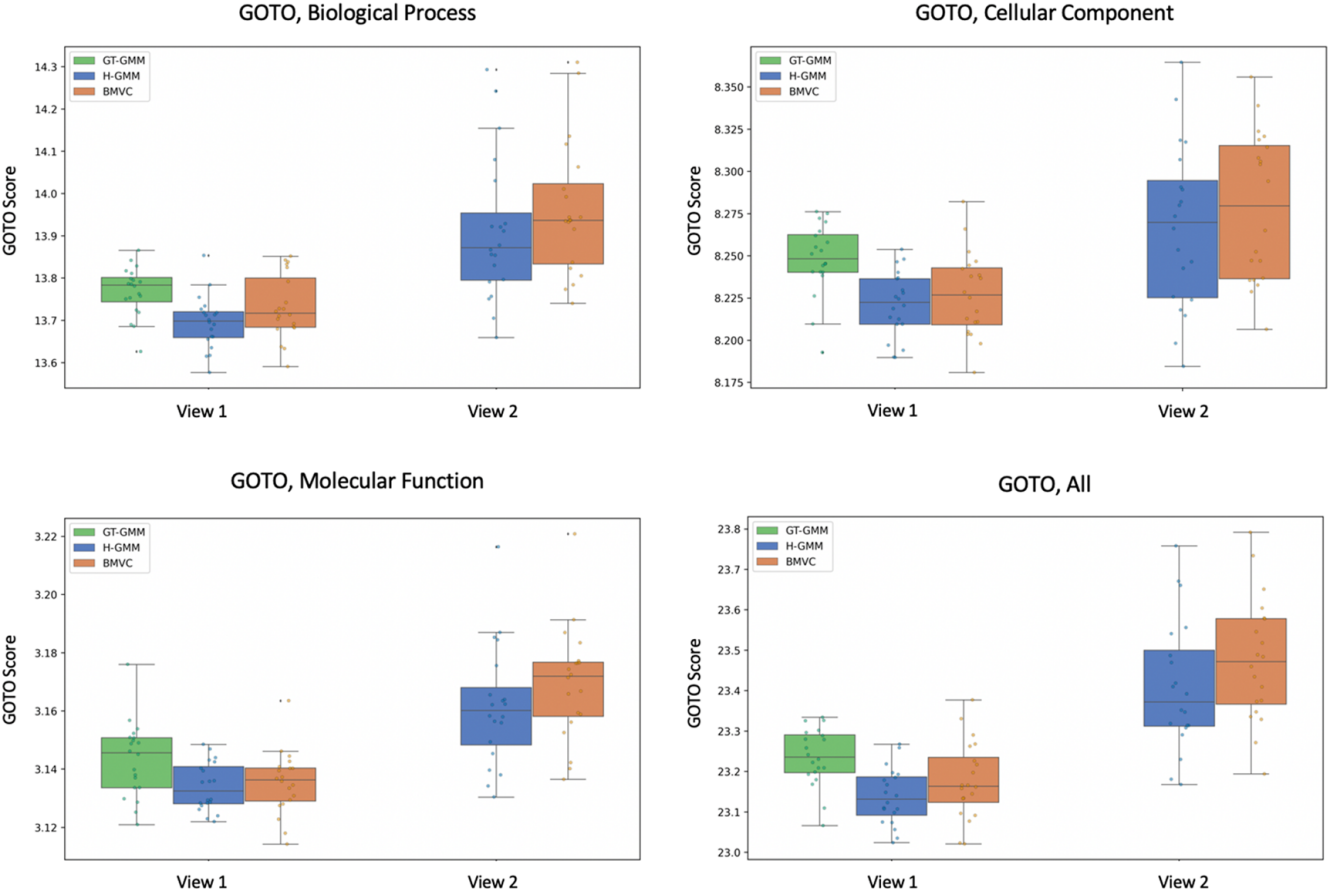
Bayesian Multi-View Clustering analyses of the pseudo-simulated data better capture the underlying biological structure than Gaussian mixture models, as demonstrated here using the Gene Ontology Term Overlap statistic as a measure of biological homogeneity. Notably, ground truth clusterings of the noiseless data (in green, GT-GMM) can only be observed in the first view of the pseudo-simulated data, which contains no duplication. These clusterings achieve the highest scores, which supports the use of GOTO as a measure of biological homogeneity when measuring the performance of BMVC.

In our preliminary pseudo-simulated experiment, we found that BMVC clusterings had a higher average ARI than H-GMM clusterings ( Figure 6), where we took ground truth to be the clustering of the true expression data. Correspondingly, BMVC GOTO scores ( Figure 7) were higher on average than those produced by H-GMM in both views and in all GO conditions, and, thus, they were closer in the first view to the GOTO scores produced by the reference GT-GMM clustering. Note that the duplication of entities in the second view of our pseudo-simulated data changed the baseline distribution of the data, and as such there was no ground truth; thus, we were not able to generate GOTO scores for GT-GMM in the second view ( Figure 7), nor did we compute ARI in the second view ( Figure 6). The correlation between an improvement in the ARI and an improvement in GOTO score suggests that GOTO is a reasonable, if approximate, measure of true biological homogeneity. Given this assumption, an improvement in GOTO by BMVC over H-GMM when applied to the true biological data would suggest that BMVC is able to successfully leverage the known inter-view relationships to produce an improved multi-view clustering.

**Figure 8.**
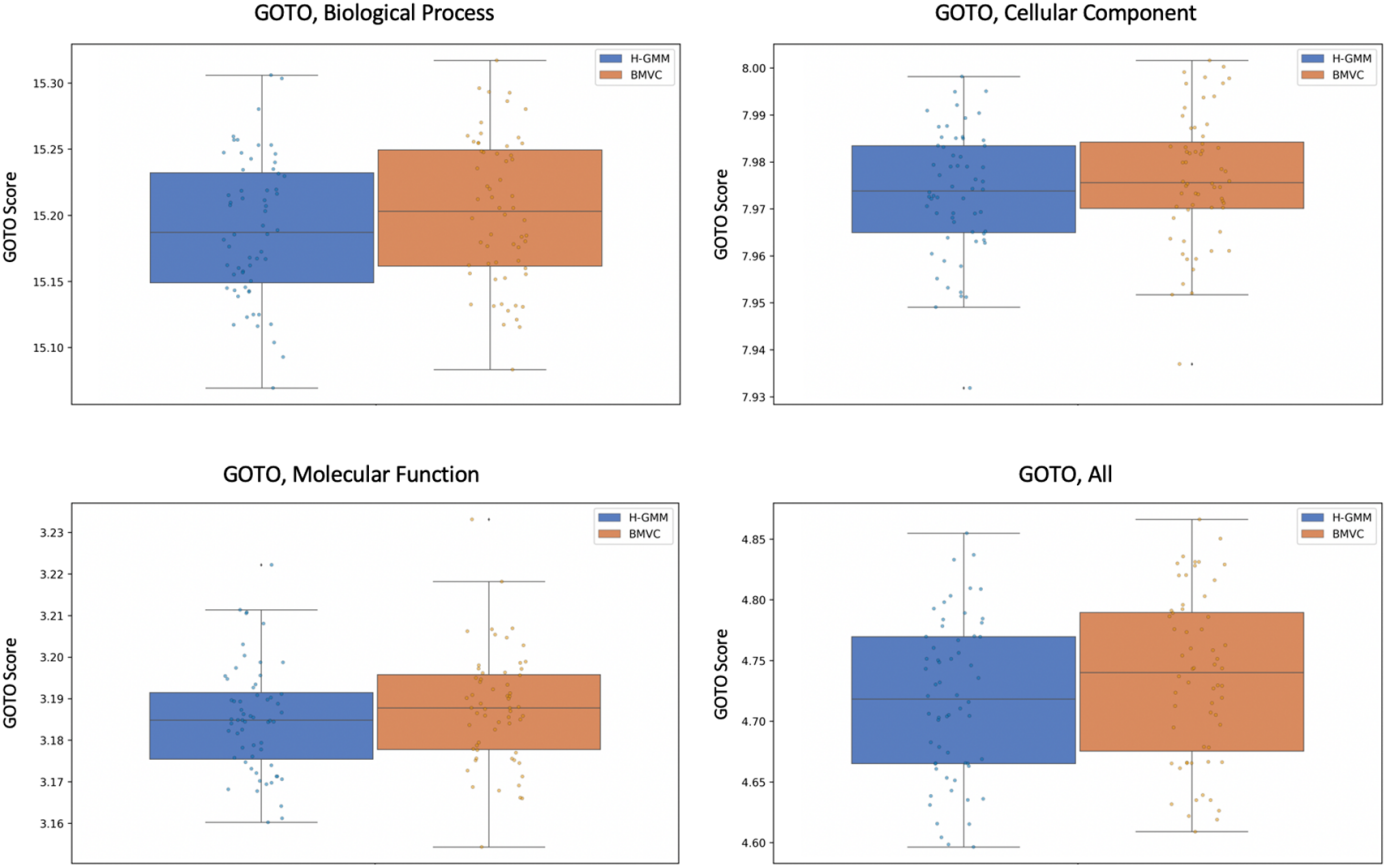
GOTO scores in each Gene Ontology category show that the average biological homogeneity of BMVC clusterings across views is higher than in clusterings produced by the Gaussian mixture models.

We ran BMVC and the independent GMMs on the processed expression and methylation datasets together, each with 60 random but identical initializations and no added noise (in contrast to our pseudo-simulations). Figure 8 compares the performance of BMVC and H-GMM in terms of average GOTO scores across the views and demonstrates that BMVC improves the overall biological homogeneity of the joint clustering.

Notably, the improvement in the methylation view when using BVMC is larger than in the expression view ( Table 3), which is consistent with the observation that methylation data is generally noisier ^25^ and so may benefit more from transfer learning. GOTO scores of methylation clusterings may also increase more when informed by gene expression clusterings because GOTO is defined with respect to gene products rather than epigenetic processes. This could explain why biasing gene expression clusterings to more closely resemble the structure found in the epigenetic data does not increase the GOTO score in the expression view as much.

**Table 3.** Average Gene Ontology Term Overlap scores, measured in four Gene Ontology categories: Biological Process (BP), Cellular Component (CC), Molecular Function (MF), and all GO terms combined (All).

	Expression		Methylation	
	H-GMM	BMVC	H-GMM	BMVC
BP	6.0893	**6.0936**	8.7330	**8.7513**
CC	5.5989	**5.6015**	5.2022	**5.2051**
MF	1.9272	1.9272	2.1458	**2.1512**
All	12.6879	**12.6941**	15.4830	**15.5088**

We performed GSEA for biological processes of the BMVC gene expression clusters over all trials alongside the corresponding H-GMM clusters using KEGG and Reactome Canonical Pathway genesets from MSigDB, ^28^^,^^30^ using the Benjamini-Hochberg correction at a FDR threshold of 0.1. We compiled a list of the enrichments found by BMVC but not the GMMs in each trial. We then compiled the list of enrichments found by the GMMs but not by BMVC in each trial; we counted the occurrences of the enrichments in each list and subtracted the counts of the second list from the first in order to control for random effects due to initialization. Finally, we examined the top scoring enrichments that were found in three or more remaining BMVC trials in order to determine what biological effects were most often recovered by BMVC clusterings but not by the independent Gaussian mixture models.

We found that by incorporating the dependency between the views, we were more likely to recover enrichments consistent with previous findings of the role of methylation in alternative splicing in breast cancers as well as and in regulation of pre-mRNA splicing (KEGG_SPLICEOSOME, REACTOME_MRNA_SPLICING, REACTOME_PROCESSING_OF_CAPPED_INTRON_CON-TAINING_PRE_MRNA). ^31^^,^^32^ We were also more likely to recover enrichments of the S phase (REACTOME_S_PHASE), which is consistent with previous work suggesting the existence of a methylation ‘loss clock’ which links late S-phase genome replication with a reduction of tumor methylation and an accumulation of methylation errors in breast cancer tumors. ^33^ The top scoring enrichments also included processes related to G-protein coupled receptor signaling, which have been repeatedly implicated in tumorigenesis ^34^ and in particular secretin receptors (REACTOME_SIGNALING_BY_GPCR, REACTOME_CLASS_B_2_SECRETIN_FAMILY_RECEPTORS) which have previously been shown to be hypermethylated and downregulated in breast cancer tissues ^35^ and to be regulators of tumor cell proliferation. We additionally found three overrepresented enrichments for pyrimidine metabolism, the lysosomal system and basal cell carcinoma development (REACTOME_PYRIMIDINE_METABOLISM, KEGG_LYSOSOME and KEGG_BASAL_CELL_CARCINOMA) for which there are clearly documented relationships to cancer progression in the existing literature. ^36^^–^^38^ However, we found limited or no clear evidence for known mechanisms by which these processes are regulated by methylation. For example, previous findings ^38^ demonstrate that patients with frequent basal cell carcinomas may be more susceptible to the development of breast cancers due to germ-line defects in DNA repair genes. Other work stipulates that epigenetic silencing of said DNA repair genes could be a factor in tumorigenesis in cancers, ^39^ but further investigation is required to determine whether there is a specific epigenetic link between basal cell carcinomas and breast cancer development. Separately, recent work suggests that epigenetic mechanisms may underlie lysosomal storage disorders, ^40^ but that such a claim warrants further investigation. A different possible interpretation of these enrichments is that some of them represent gene expression mechanisms that are not directly affected by cis-regulatory methylation, but rather are much more identifiable in our clusterings when we account for the effects of methylation regulatory processes.

**Figure 9.**
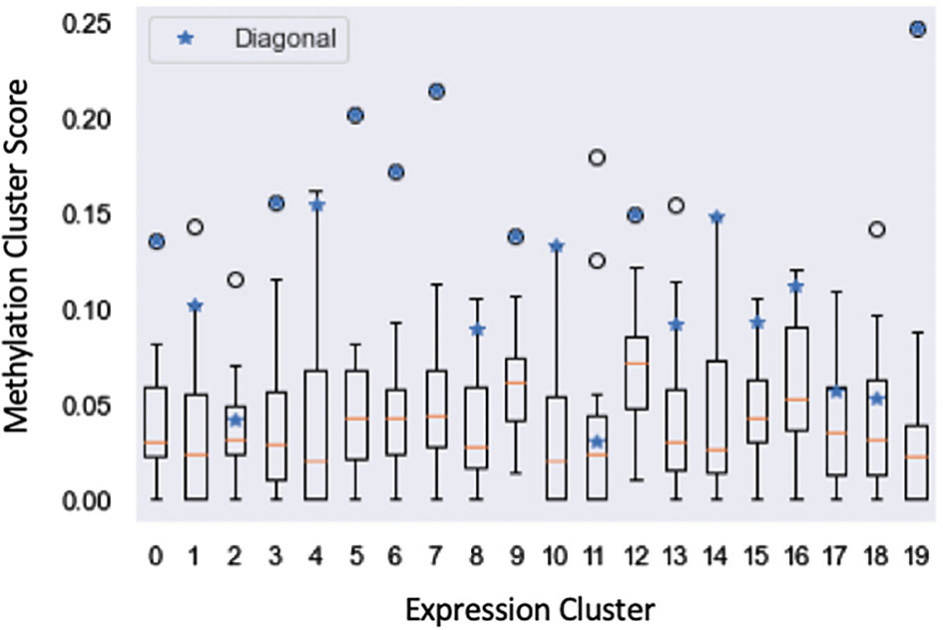
Structural affinity box plot showing affinity scores of the Bayesian Multi-view Clustering methylation clusters (y-axis) with respect to each expression cluster (x-axis). Blue stars denote the structural affinity scores of the methylation cluster respectively paired with each expression cluster.

We further examined the clustering run that achieved the highest average biological process GOTO score through the lens of the structural affinity metric used for the previous public health analysis ( Figure 9). Among the enriched clusters with high structural affinity scores, we found enrichment for immune system processes, interferon signaling and immune cytokine signaling (cluster 19, *e.g.*: REACTOME_IMMUNE_SYSTEM, REACTOME_INTERFERON_ALPHA_BETA_SIGNALING, REACTOME_CYTOKINE_SIGNALING_IN_IMMUNE_SYSTEM), which previous work shows are processes significantly correlated with genomic demethylation across tumour types. ^41^ Notably, cluster 19 was also enriched for KEGG_LYSOSOME, lending further credence to the potential for the regulation of lysosomal processes via methylation in breast cancer and potentially in lysosomal storage disorders in general as previously suggested. ^40^ We found further evidence for lipid-related processes in cluster 6 including enrichment for the peroxisome proliferator-activated receptor pathway (KEGG_PPAR_SIGNALING_PATHWAY), which is critical to metabolic and lipid-related processes and has also been found to regulate and be regulated by epigenetic processes including by DNA methyltransferases. ^42^

Inspection of low-affinity clusters was also informative. Cluster 2 was enriched for KEGG_PRIMARY_IMMUNODEFICIENCY and KEGG_HEMATOPOIETIC_CELL_LINEAGE. A class of rare disorders, primary immunodeficiency diseases (PIDs) are often treated with hematopoietic cell transplants and increase the risk of a cancer diagnosis. ^43^ While secondary immunodeficiencies may be caused by environmental or epigenetic factors, PIDs are caused by genetic mutations, and thus genes implicated in PIDs are much less likely to be regulated by methylation in this context. ^44^ The low structural affinity of cluster 2 in the BMVC clustering supports this claim and is another example of the way in which BMVC is able to provide a highly interpretable result with inferred relationships that are useful for hypothesis generation. Gene expression cluster 11 was not enriched for any terms, which might suggest that the corresponding methylation cluster represents an effect that does not functionally cis-regulate expression at the loci measured in this study; as such, this could warrant further investigation.

## Discussion

An assumption of one-to-one relationships between entities often does not sufficiently represent structure across domains in multi-view applications. We show that BMVC can be used to effectively capture latent structure in multi-view data without imposing a one-to-one assumption. BMVC is the first fully Bayesian approach to accomplish this goal, and as such it provides a flexible framework that allows many other possible configurations of the model that may be appropriate for specific applications, including user’s choice of priors on latent variables and base distributions for a given data type. Furthermore, our applications in public health and genomics highlight the ability of BMVC to generate natural interpretations of latent multi-view structure and to support mechanistic multi-view hypothesis generation. This shows promise for multi-view applications that commonly require formulation of novel hypotheses such as drug discovery and patient diagnosis.

The experiments presented here also demonstrate that our alternating inference procedure, in combination with gradient-based methods applied to the pseudo-likelihood, yields a relatively efficient inference approach capable of estimation with large numbers of entities and many clusters and relationships. Scalability will become increasingly important as the diversity and quantity of multi-view data increases across domains.

Some limitations of BMVC are worth noting. One is its reliance on *a priori* known relationships encoded by the user-specified matrix *R.* Care must be taken to ensure that the relationships encoded by *R* reflect real and high-quality relationships, *e.g.* by filtering to relationships or entities that are correlated with particular outcomes or that have higher variance as we did in our biological analysis. Additionally, extra work may be needed to account for different kinds of real-world relationships. While using known relationships is likely to be a more robust approach than an attempt to automatically infer such relationships as a pre-processing step (such as in CMMVC ^12^), future work might include estimation or refinement of entity relationships as part of the clustering process itself. Another limitation of BMVC is that it uses hard cluster assignments, thus imposing discrete assumptions of cluster membership. The hard assignments directly determine the impact of *ϕ* on the overall clustering, and also contribute to the efficiency of the inference procedure proposed here. An effort to incorporate uncertainty into the estimation of cluster assignments by using soft expectation maximization could further improve the accuracy and robustness of the multi-view clustering.

There are also significant opportunities for extensions and further work in complex multi-view structure learning. It will continue to be important to ensure that these models are interpretable, especially in critical settings such as the clinic. Here, we presented *post-hoc* interpretations of the inter-view cluster relationships by visualizing structural affinity scores. Future work could focus on Bayesian approaches that explicitly consider more fine-grained representations of the relationships in the data, *e.g.* by including a different latent variable for every pair of clusters between two views. Further, future approaches could include efforts to account for continuous structure beyond clusters, such as through factorization, and further complex dependencies between these structures, such as via hierarchical or tree-based models. We expect that flexible approaches like BMVC that can incorporate increasingly complex assumptions will continue to improve the application of multi-view models in real-world contexts.

## Data Availability

Data from The Cancer Genome Atlas used in this work is available publicly through the Genomic Data Commons at https://gdc.cancer.gov/about-data/publications/brca_2012. The public health data surveying individuals and households in Puerto Rico following Hurricane Maria is available under the Creative Commons Attribution 3.0 license and can be found at https://github.com/c2-d2/pr_mort_official . The source code for BMVC can be found on GitHub at https://github.com/bshapiro/bmvc-paper . The archived source code at time of publication is available at https://doi.org/10.5281/zenodo.7250670. ^45^ An implementation of GOTO score computation for both expression and methylation data in Python can be found at https://github.com/bshapiro/gotopy. The archived source code at time of publication is available at https://doi.org/10.5281/zenodo.7250678. ^46^ All software is distributed under the BSD 3-Clause License. Any additional information required to replicate any results reported in this paper is available from the lead contact upon request.

## References

[1] Shen R Olshen AB Ladanyi M : Integrative clustering of multiple genomic data types using a joint latent variable model with application to breast and lung cancer subtype analysis. *Bioinformatics.* 2009;25(22):2906–2912. 10.1093/bioinformatics/btp543 19759197 PMC2800366

[2] Shen R Wang S Mo Q : Sparse integrative clustering of multiple omics data sets. *Ann. Appl. Stat.* 2013;7(1):269–294. 10.1214/12-AOAS578 24587839 PMC3935438

[3] Mo Q Wang S Seshan VE : Pattern discovery and cancer gene identification in integrated cancer genomic data. *Proc. Natl. Acad. Sci. U. S. A.* 2013;110(11):4245–4250. 10.1073/pnas.1208949110 23431203 PMC3600490

[4] Mo Q Shen R Guo C : A fully Bayesian latent variable model for integrative clustering analysis of multi-type omics data. *Biostatistics.* 2018;19(1):71–86. 10.1093/biostatistics/kxx017 28541380 PMC6455926

[5] Kirk P Griffin JE Savage RS : Bayesian correlated clustering to integrate multiple datasets. *Bioinformatics.* 2012;28(24):3290–3297. 10.1093/bioinformatics/bts595 23047558 PMC3519452

[6] Lock EF Dunson DB : Bayesian consensus clustering. *Bioinformatics.* 2013;29(20):2610–2616. 10.1093/bioinformatics/btt425 23990412 PMC3789539

[7] Gabasova E Reid J Wernisch L : Clusternomics: Integrative context-dependent clustering for heterogeneous datasets. *PLoS Comput. Biol.* 2017;13(10):1–29. 10.1371/journal.pcbi.1005781 PMC5658176 29036190

[8] Brett D Pospisil H Valcárcel J : Alternative splicing and genome complexity. *Nat. Genet.* 2002;30(1):29–30. 11743582 10.1038/ng803

[9] Lee DD Seung HS : Algorithms for non-negative matrix factorization. *Adv. Neural Inf. Proces. Syst.* 2001;556–562.

[10] Liu J Wang C Gao J : Multi-view clustering via joint nonnegative matrix factorization. *Proceedings of the 2013 SIAM International Conference on Data Mining, SDM 2013.* 2013; pp.252–260.

[11] Cheng W Guo Z Zhang X : CGC: A flexible and robust approach to integrating co-regularized multi-domain graph for clustering. *ACM Trans. Knowl. Discov. Data.* 2016;10(4):1–27. 10.1145/2903147 29081726 PMC5658064

[12] Yu H Xiong J Zhang X : Multi-view clustering by exploring complex mapping relationship between views. 2020.

[13] Liu J Teng S Fei L : A novel consensus learning approach to incomplete multi-view clustering. *Pattern Recogn.* 2021;115:107890. 10.1016/j.patcog.2021.107890

[14] Yin J Sun S : Incomplete multi-view clustering with cosine similarity. *Pattern Recogn.* 2022;123:108371. 10.1016/j.patcog.2021.108371

[15] Li SY Jiang Y Zhou ZH : Partial multi-view clustering. *Proceedings of the National Conference on Artificial Intelligence.* 2014; vol.3: pp.1968–1974.

[16] Zhu P Yao X Wang Y : Latent Heterogeneous Graph Network for Incomplete Multi-View Learning. *IEEE Transactions on Multimedia.* 2022;1–13.

[17] Ranganath R Gerrish S Blei DM : Black box variational inference. *J. Mach. Learn. Res.* 2014;33:814–822.

[18] Kingma DP Ba JL : Adam: A method for stochastic optimization. *3rd International Conference on Learning Representations, ICLR 2015 - Conference Track Proceedings.* 2015; pp.1–15.

[19] Kingma DP Welling M : Auto-encoding variational bayes. *2nd International Conference on Learning Representations, ICLR 2014 - Conference Track Proceedings.* 2014; no.Ml: pp.1–14.

[20] Koller D Friedman N : *Probabilistic graphical models: principles and techniques.* MIT Press;2009.

[21] Kishore N Marqués D Mahmud A : Mortality in Puerto Rico after Hurricane Maria. *N. Engl. J. Med.* 2018;379(2):162–170. 10.1056/NEJMsa1803972 29809109

[22] O’Connor NR Dougherty M Harris PS : Survival after dialysis discontinuation and hospice enrollment for ESRD. *Clin. J. Am. Soc. Nephrol.* 2013;8(12):2117–2122. 10.2215/CJN.04110413 24202133 PMC3848402

[23] Lin CM Davidson TM Ancoli-Israel S : Gender differences in obstructive sleep apnea and treatment implications. 2008. 10.1016/j.smrv.2007.11.003 PMC2642982 18951050

[24] Perez TA Castillo EG Ancochea J : Sex differences between women and men with COPD: A new analysis of the 3CIA study. *Respir. Med.* 2020;171:106105. 10.1016/j.rmed.2020.106105 32858497

[25] Koboldt DC Fulton RS McLellan MD : Comprehensive molecular portraits of human breast tumours. *Nature.* 2012;490(7418):61–70. 23000897 10.1038/nature11412 PMC3465532

[26] Zhou W Laird PW Shen H : Comprehensive characterization, annotation and innovative use of Infinium DNA methylation BeadChip probes. *Nucleic Acids Res.* 2017;45(4):e22. 10.1093/nar/gkw967 27924034 PMC5389466

[27] Mistry M Pavlidis P : Gene Ontology term overlap as a measure of gene functional similarity. *BMC Bioinformatics.* 2008;9:1–11. 18680592 10.1186/1471-2105-9-327 PMC2518162

[28] Subramanian A Tamayo P Mootha VK : Gene set enrichment analysis: A knowledge-based approach for interpreting genome-wide expression profiles. *Proc. Natl. Acad. Sci. U. S. A.* 2005;102(43):15545–15550. 10.1073/pnas.0506580102 16199517 PMC1239896

[29] Geeleher P Hartnett L Egan LJ : Gene-set analysis is severely biased when applied to genome-wide methylation data. *Bioinformatics.* 2013;29(15):1851–1857. 10.1093/bioinformatics/btt311 23732277

[30] Liberzon A Birger C Thorvaldsdóttir H : The Molecular Signatures Database Hallmark Gene Set Collection. *Cell Systems.* 2015;1(6):417–425. 10.1016/j.cels.2015.12.004 26771021 PMC4707969

[31] Pant D Narayanan SP Vijay N : Hypoxia-induced changes in intragenic DNA methylation correlate with alternative splicing in breast cancer. *J. Biosci.* 2020;45(1):1–24. 10.1007/s12038-019-9977-0 31965981 PMC7117958

[32] Maunakea AK Chepelev I Cui K : Intragenic DNA methylation modulates alternative splicing by recruiting MeCP2 to promote exon recognition. *Cell Res.* 2013;23(11):1256–1269. 10.1038/cr.2013.110 23938295 PMC3817542

[33] Batra RN Lifshitz A Vidakovic AT : DNA methylation landscapes of 1538 breast cancers reveal a replication-linked clock, epigenomic instability and cis-regulation. *Nat. Commun.* 2021;12(1):1–13. 10.1038/s41467-021-25661-w 34518533 PMC8437946

[34] Lappano R Jacquot Y Maggiolini M : GPCR modulation in breast cancer. *Int. J. Mol. Sci.* 2018;19(12). 10.3390/ijms19123840 30513833 PMC6321247

[35] Kang S Kim B Kang HS : SCTR regulates cell cycle-related genes toward anti-proliferation in normal breast cells while having pro-proliferation activity in breast cancer cells. *Int. J. Oncol.* 2015;47(5):1923–1931. 10.3892/ijo.2015.3164 26397240

[36] Wang W Cui J Ma H : Targeting Pyrimidine Metabolism in the Era of Precision Cancer Medicine. *Front. Oncol.* 2021;11(May):1–17. 10.3389/fonc.2021.684961 PMC8194085 34123854

[37] Tang T Yang Z y Wang D : The role of lysosomes in cancer development and progression. *Cell Biosci.* 2020;10(1):1–18. 33292489 10.1186/s13578-020-00489-x PMC7677787

[38] Cho HG Kuo KY Li S : Frequent basal cell cancer development is a clinical marker for inherited cancer susceptibility. *JCI insight.* 2018;3(15). 10.1172/jci.insight.122744 30089731 PMC6129130

[39] Lahtz C Pfeifer GP : Epigenetic changes of DNA repair genes in cancer. *J. Mol. Cell Biol.* 2011;3(1):51–58. 10.1093/jmcb/mjq053 21278452 PMC3030973

[40] Hassan S Sidransky E Tayebi N : The role of epigenetics in lysosomal storage disorders: Uncharted territory. *Mol. Genet. Metab.* 2017;122(3):10–18. 10.1016/j.ymgme.2017.07.012 28918065

[41] Jung H Kim HS Kim JY : DNA methylation loss promotes immune evasion of tumours with high mutation and copy number load. *Nat. Commun.* 2019;10(1):1–12. 31537801 10.1038/s41467-019-12159-9 PMC6753140

[42] Porcuna J Mínguez-Martínez J Ricote M : The ppar *α* and ppar *γ* epigenetic landscape in cancer and immune and metabolic disorders. *Int. J. Mol. Sci.* 2021;22(19). 10.3390/ijms221910573 34638914 PMC8508752

[43] Mortaz E Tabarsi P Mansouri D : Cancers related to immunodeficiencies: Update and perspectives. *Front. Immunol.* 2016;7(SEP):1–13. 10.3389/fimmu.2016.00365 27703456 PMC5028721

[44] Martínez-Cano J Campos-Sánchez E Cobaleda C : Epigenetic Priming in Immunodeficiencies. *Front. Cell Dev. Biol.* 2019;7(July). 10.3389/fcell.2019.00125 31355198 PMC6635466

[45] Shapiro BD : bshapiro/bmvc-paper: 0.1.0. Oct. 2022. 10.5281/zenodo.7250670

[46] Shapiro BD : bshapiro/gotopy: 0.1.0. Oct. 2022. 10.5281/zenodo.7250678

