# Supplementary material for: Bayesian Multi-View Clustering given complex inter-view structure
Source: F1000Res. 2024 Feb 29;11:1460. Originally published 2022 Dec 9. [Version 2] doi: 10.12688/f1000research.126215.2 (PMC10940850; doi:10.12688/f1000research.126215.2)
Supplement: Peer review history

# Reviewer report: Reviewer response for version 2

**DOI:** 10.5256/f1000research.162164.r251743

**Published:** 2024-03-14

**Authors:** Max Moldovan

**Affiliations:** 1 The University of Adelaide, Adelaide, South Australia, Australia

The authors have appropriately revised the manuscript.

Is the rationale for developing the new method (or application) clearly explained?

Yes

Is the description of the method technically sound?

Yes

Are the conclusions about the method and its performance adequately supported by the findings presented in the article?

Yes

If any results are presented, are all the source data underlying the results available to ensure full reproducibility?

Yes

Are sufficient details provided to allow replication of the method development and its use by others?

Partly

Reviewer Expertise:

Data science

I confirm that I have read this submission and believe that I have an appropriate level of expertise to confirm that it is of an acceptable scientific standard.

# Reviewer report: Reviewer response for version 1

**DOI:** 10.5256/f1000research.138601.r220444

**Published:** 2023-11-10

**Authors:** Max Moldovan

**Affiliations:** 1 The University of Adelaide, Adelaide, South Australia, Australia

The authors presented a Bayesian Multi-View Clustering (BMVC) method that allows identifying clusters in multi-view systems. Such systems are especially common in biology, being often characterised by multiple distinct but inter-dependent information layers, such as gene expression, DNA methylation and proteomic profiles. As presented by the authors, the proposed BMVC approach is able to utilise between-views relationships when recovering clustering within each individual view. The authors have tested the method with simulated data and based on two different empirical datasets. The authors have also provided Python codes for the method implementation on the dedicate github.

 The method is practically attractive and can be used in various data-rich fields. While reading the paper, I had some remaining questions regarding the method. Firstly, model estimation requires maximisation of a complex function containing multiple local optima. What is the variation of resulted clustering outcomes due to alternative attained local optima, and especially with respect to the global optimum?

 Secondly, the authors recognise that their approach relies on hard cluster assignments (i.e. each node can belong to a single cluster only) and list this as a method limitation. How hard would it be to implement fuzzy cluster assignments in the suggested method? Would it be an extension of the proposed method, or perhaps a new method with outcomes greatly different for hard and fuzzy cluster assignments even if applied to the same dataset?

 Finally, how sensitive clustering outcomes are to misspecification of R matrix (the matrix that codes between-view relationships)?

 To summarise, the authors have presented a practically relevant and implementation promising method for assigning clusters within multi-view systems. The authors have provided Python codes for the method implementation, which would hopefully be replaced by more formal and better documented Python and R packages.

Is the rationale for developing the new method (or application) clearly explained?

Yes

Is the description of the method technically sound?

Yes

Are the conclusions about the method and its performance adequately supported by the findings presented in the article?

Yes

If any results are presented, are all the source data underlying the results available to ensure full reproducibility?

Yes

Are sufficient details provided to allow replication of the method development and its use by others?

Partly

Reviewer Expertise:

Data science

I confirm that I have read this submission and believe that I have an appropriate level of expertise to confirm that it is of an acceptable scientific standard, however I have significant reservations, as outlined above.

# Reviewer report: Reviewer response for version 1

**DOI:** 10.5256/f1000research.138601.r158015

**Published:** 2023-01-16

**Authors:** Sara Mostafavi

**Affiliations:** 1 Paul G. Allen School of Computer Science and Engineering, University of Washington, Seattle, WA, USA

The authors present a Bayesian multi-view clustering methods called BMVC that can be applied to multi-modal data. The methods presented is sound, and has novel statistical properties (such as estimating uncertainty, and modifying model to incorporate different types of prior depending on the input data types). BMVC is applied and carefully evaluated in three very different settings, 1) simulation data, 2) public health data, and 3) gene expression paired with DNA methylation data. The authors do a nice job in demonstrating that BMVC leads to improved results over the baseline clustering approaches. 

 I specifically appreciate the detailed analysis the authors performed on the cancer gene expression + DNA methylation data. I have no major comments to be addressed.

Is the rationale for developing the new method (or application) clearly explained?

Yes

Is the description of the method technically sound?

Yes

Are the conclusions about the method and its performance adequately supported by the findings presented in the article?

Yes

If any results are presented, are all the source data underlying the results available to ensure full reproducibility?

Yes

Are sufficient details provided to allow replication of the method development and its use by others?

Yes

Reviewer Expertise:

Computational Biology; Gene regulation

I confirm that I have read this submission and believe that I have an appropriate level of expertise to confirm that it is of an acceptable scientific standard.
